# Condition-specific changes in gaze allocation following gaze-triggered interpersonal-distance outcomes

**DOI:** 10.3758/s13414-026-03322-8

**Published:** 2026-08-31

**Authors:** Canling An, Soichiro Matsuda

**Affiliations:** 1 Doctoral Program in Psychology, Graduate School of Comprehensive Human Sciences, University of Tsukuba, 1-1-1 Tennodai, Tsukuba, 305-8573 Japan; 2 Institute of Human Sciences, University of Tsukuba, Ibaraki, Japan

**Keywords:** Eye movements and visual attention, Eye Movements: Cognitive, Attention in learning

## Abstract

**Abstract:**

Interpersonal distance is a fundamental social cue, yet it remains unclear whether repeated exposure to gaze-triggered approach or recede animations is associated with subsequent gaze allocation. This study examined whether such gaze-triggered changes in apparent interpersonal distance were associated with changes in dwell-time allocation toward face stimuli. Fifty adults completed two within-subject blocks (Approach vs. Recede), each consisting of Baseline, Training, and Test phases. During Training, fixating a designated face for a gaze-duration threshold triggered a predefined approach or recede animation that proceeded independently of subsequent gaze behavior, while the comparison face remained static. Gaze allocation was operationalized as the proportion of dwell time directed toward the face associated with the gaze-triggered animation and was assessed during the static Baseline and Test phases. Linear mixed-effects analyses revealed a significant Condition × Phase interaction: dwell-time proportion decreased from Baseline to Test following gaze-triggered approach animations, whereas no reliable group-level change was observed following recede animations. These findings indicate that repeated exposure to gaze-triggered changes in apparent interpersonal distance is associated with systematic changes in subsequent gaze allocation. The approach-related reduction suggests that gaze-triggered distance changes may redistribute later gaze allocation rather than simply increasing looking toward the stimulus associated with approach. The observed effects do not imply subjective evaluation, value acquisition, or a specific learning mechanism. This paradigm provides a controlled approach for examining how gaze-triggered interpersonal-distance changes are associated with later attentional allocation.

**Open practices statement:**

De-identified trial-level data and analysis code for this study are available via the Open Science Framework: https://doi.org/10.17605/OSF.IO/EG4B6. This study was not preregistered.

**Supplementary Information:**

The online version contains supplementary material available at https://doi.org/10.3758/s13414-026-03322-8.

## Introduction

In everyday social interactions, directing gaze toward another person is often followed by social engagement, including changes in interpersonal distance. Although approaching and receding movements provide information about distance changes, it remains unclear whether repeated exposure to gaze-triggered changes in apparent interpersonal distance is related to later patterns of gaze allocation.

Previous research has shown that learned associations can influence visual attention, so that stimuli linked to reward continue to attract attention even when they are no longer task relevant (Anderson et al., 2011; Anderson, 2016; Watson et al., 2020). These effects have been observed for outcomes such as monetary rewards and facial expressions, which carry explicit social or motivational meaning (Anderson, 2015; Anderson et al., 2011).

In parallel, studies of spatial and distance-related cues indicate that approaching or nearby stimuli can modulate attentional processing. Behavioral work shows faster orienting responses to approaching stimuli (Chen et al., 2012), and electrophysiological studies suggest that stimuli in near or peripersonal space influence neural markers of attention (Kasai et al., 2003; Martin et al., 2021; Veranic et al., 2025). However, these lines of research have typically focused on perceptual or motor responses, rather than sustained gaze allocation measured after repeated exposure to distance-related outcomes. Related work on interpersonal distance has also relied on behavioral paradigms such as the stop-distance task (Hayduk, 1983; Perry et al., 2013) and the approach–avoidance task (Rinck & Becker, 2007). These paradigms primarily examine how individuals regulate distance or action tendencies, rather than how repeated exposure to distance-related outcomes is associated with later gaze allocation. Importantly, later gaze allocation need not simply mirror behavioral responses such as approach, avoidance, or distance regulation. A person may withdraw from an interaction while still monitoring the relevant social stimulus, or may shift gaze away after repeated exposure to an approach-related event. Taken together, prior work suggests that both learned outcome associations and distance-related perceptual cues can influence attention. What remains unclear is whether repeated exposure to distance-related outcomes is associated with subsequent gaze allocation when stimuli are later presented without motion.

To examine this question, the present study builds on prior work using gaze-triggered feedback while introducing a more temporally discrete implementation. Previous studies have shown that gaze-contingent paradigms, in which stimulus changes are continuously coupled with gaze position and cease when gaze is withdrawn, can influence subsequent patterns of visual attention (Matsuda et al., 2019; Wang et al., 2020). This temporally discrete structure may capture one constrained aspect of everyday social interactions, in which sustained gaze can mark a transition point after which subsequent interpersonal events unfold without being continuously dependent on ongoing gaze. In this sense, a gaze-triggered design allows the initiating role of gaze to be examined while avoiding continuous gaze–stimulus coupling during the outcome itself.

In the present study, we instead use the term gaze-triggered to describe a mechanism in which a predefined stimulus change is initiated once a gaze-duration threshold is reached and then proceeds independently of subsequent gaze behavior. This design reduces the continuous coupling between gaze position and stimulus change, allowing prior gaze-triggered outcomes to be examined without ongoing gaze–stimulus linkage. By implementing approach and recede as gaze-triggered changes in facial image size during training, and by assessing gaze allocation in subsequent phases in which both stimuli are static, the present design provides a controlled way to examine how repeated exposure to such outcomes is associated with later patterns of gaze allocation.

The goal of the present study was to test whether gaze-triggered approach and recede outcomes are associated with subsequent changes in gaze allocation. Using a within-subjects design, we examined the effects of Condition (Approach vs. Recede) and Phase (Baseline vs. Test). In each condition, one face was designated as the face associated with the gaze-triggered approach or recede animation during training, while the other served as a static comparison face. We hypothesized that dwell-time proportion toward the outcome-associated face would change from the Baseline phase to the Test phase in the Approach condition. The Recede condition was included as a comparison condition to assess whether any observed changes in gaze allocation were specific to approach-related outcomes. Autism Spectrum Quotient (AQ) scores were included as an individual-difference factor, given prior evidence linking autistic traits to variation in gaze behavior (Klin et al., 2002). Gender and condition order were included as exploratory covariates.

## Method

### Participants

Fifty-two student participants (15 males, 37 females) from the University of Tsukuba participated (age: 18–40 years, *M* = 21, *SD* = 5). All participants were native Japanese speakers with a shared cultural background. All had normal or corrected-to-normal vision and reported no history of neurological or psychiatric conditions. All participants provided written informed consent prior to participation. The study protocol was approved by the research ethics committee of the University of Tsukuba.

Two participants were excluded because fewer than two valid trials were obtained in either the baseline or the test phase. A trial was defined as valid if the participant’s dwell time on either face exceeded 100 ms. In total, data from 50 participants were included in the final analysis. An a priori power analysis was conducted using G*Power (Faul et al., 2009) based on a comparable repeated-measures analysis of variance (ANOVA) design. The analysis estimated that 34 participants were needed to detect medium effects (α =.05, f = 0.25). To ensure stability, 52 participants were recruited. Although G*Power does not capture the hierarchical structure of mixed-effects models, it provides a conventional approximation for assessing design sensitivity.

### Apparatus

Eye movements were recorded using a Tobii X3-120 screen-based eye tracker at a sampling rate of 120 Hz. The device was mounted below the monitor and had a reported tracking accuracy of approximately 0.4–0.6° under optimal conditions. Stimuli were presented on a 1,680 × 1,050 pixel LCD monitor. Stimulus presentation and data collection were controlled using Tobii Pro Lab software (version 1.162.32461; Tobii Technology AB). All participants were tested in the same laboratory room under standard ceiling lighting. Curtains were kept closed during testing to minimize direct sunlight, and efforts were made to reduce strong shadows and screen glare. Participants were seated with their eye level aligned to the center of the screen, at a viewing distance of approximately 65 cm.

### Stimulus

Four neutral-expression face images of female models (IDs f01–f04) were selected from the AIST Facial Expression Database (Fujimura & Umemura, 2018). Female faces were chosen to maintain stimulus consistency and to reduce variability associated with sex-of-stimulus effects. These images were validated by the Affect Grid method (Russell et al., 1989). All selected images showed high agreement for the “neutral” category, and their mean valence and arousal scores were near the midpoint of the scale. Across the experiment, four face identities were used. In each block, two faces were selected from this set, with one serving as the gaze-triggered face and the other as the static comparison face. The same pair of face identities was used throughout the baseline, training, and test phases within each block. Sustained gaze on the gaze-triggered face produced Approach or Recede animations.

**Fig. 1:**
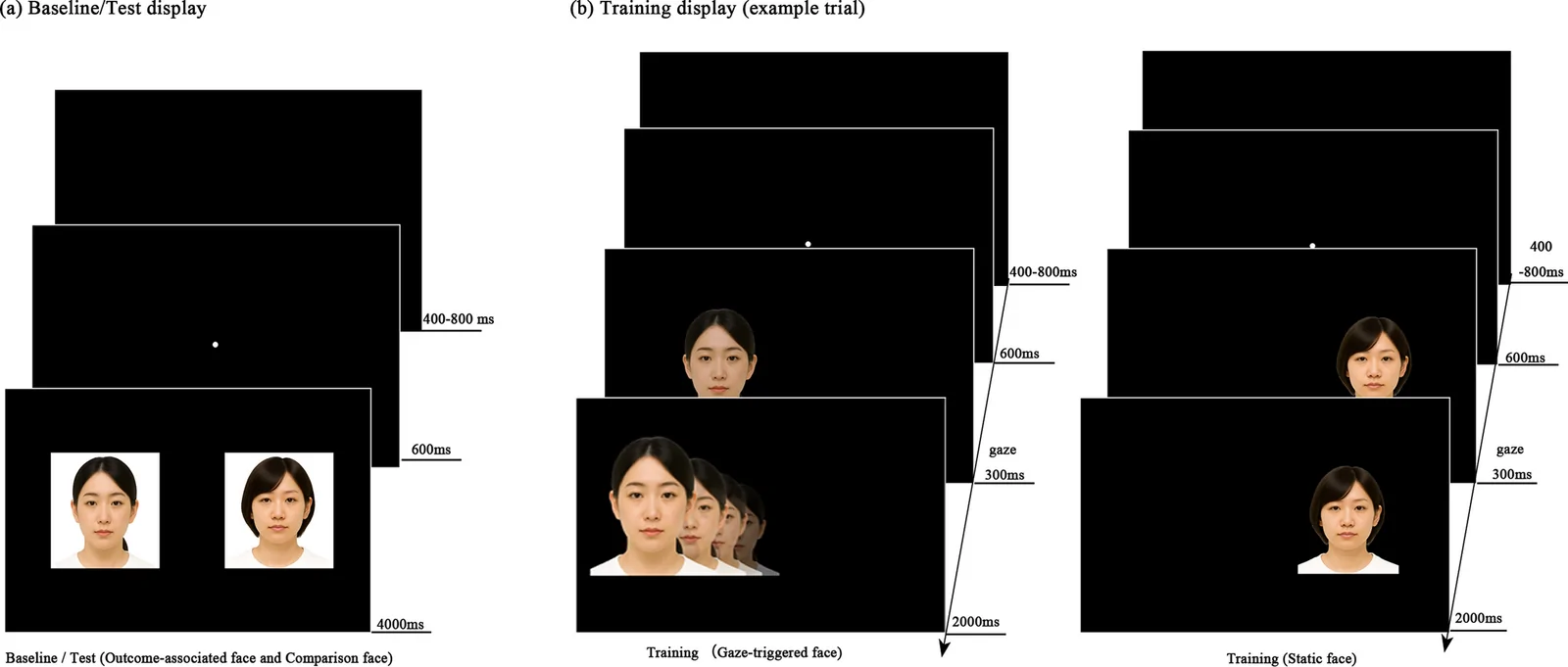
Schematic illustration of the experimental displays. **a** Baseline and Test phases. **b** Training phase (Approach condition shown as an example). The experimental stimuli were drawn from the AIST Facial Expression Database. The example faces displayed in this figure were synthetically generated by the first author for illustration purposes to avoid copyright and privacy issues

During the baseline and test phases, each trial displayed two static face images (Fig. 1a). One of the two faces served as the gaze-triggered stimulus during training. The images were presented on a uniform white background and arranged horizontally or vertically around a central fixation point. Each image measured 393 × 393 pixels (approximately 9.75° × 9.75° of visual angle). To facilitate interpretation in terms of interpersonal distance, approximate distances were estimated based on stimulus visual angles, viewing distance, and an assumed average adult face height (20–24 cm). The baseline face size corresponds to approximately 110–130 cm, with the enlarged (approach) and reduced (recede) face sizes corresponding to proportionally shorter (~70–85 cm) and longer (~300–360 cm) distances, respectively. These estimates are for interpretation only. These face images were displayed against a full-screen black background (1,680 × 1,050 pixels; RGB = 0, 0, 0), creating high contrast with the white image areas. The images were arranged to either the left and the right or above and below the central fixation point.

During the training phase, each trial presented a single face, either a gaze-triggered dynamic face or a static comparison face, positioned slightly to the left or right of the screen center (Fig. 1b). Face images were cropped to remove the surrounding background so that the size-change animation applied only to the face region. The original images were presented in the baseline and test phases because no animation was applied. When participants fixated on the gaze-triggered face for 300 ms, a 2,000-ms animation was triggered. Once initiated, the animation continued independently of subsequent gaze behavior. In the approach condition, the face advanced toward the participant, reaching approximately 635 × 635 pixels. In the recede condition, it withdrew, reaching approximately 152 × 152 pixels. This corresponded to a 6° change in visual angle, presented at a constant speed. The static face remained unchanged for the same duration. Stimulus position (left vs. right) was randomized across trials. Condition order (Approach vs. Recede) and the assignment of face identity to the gaze-triggered role were counterbalanced across participants.

### Procedures

**Fig. 2:**
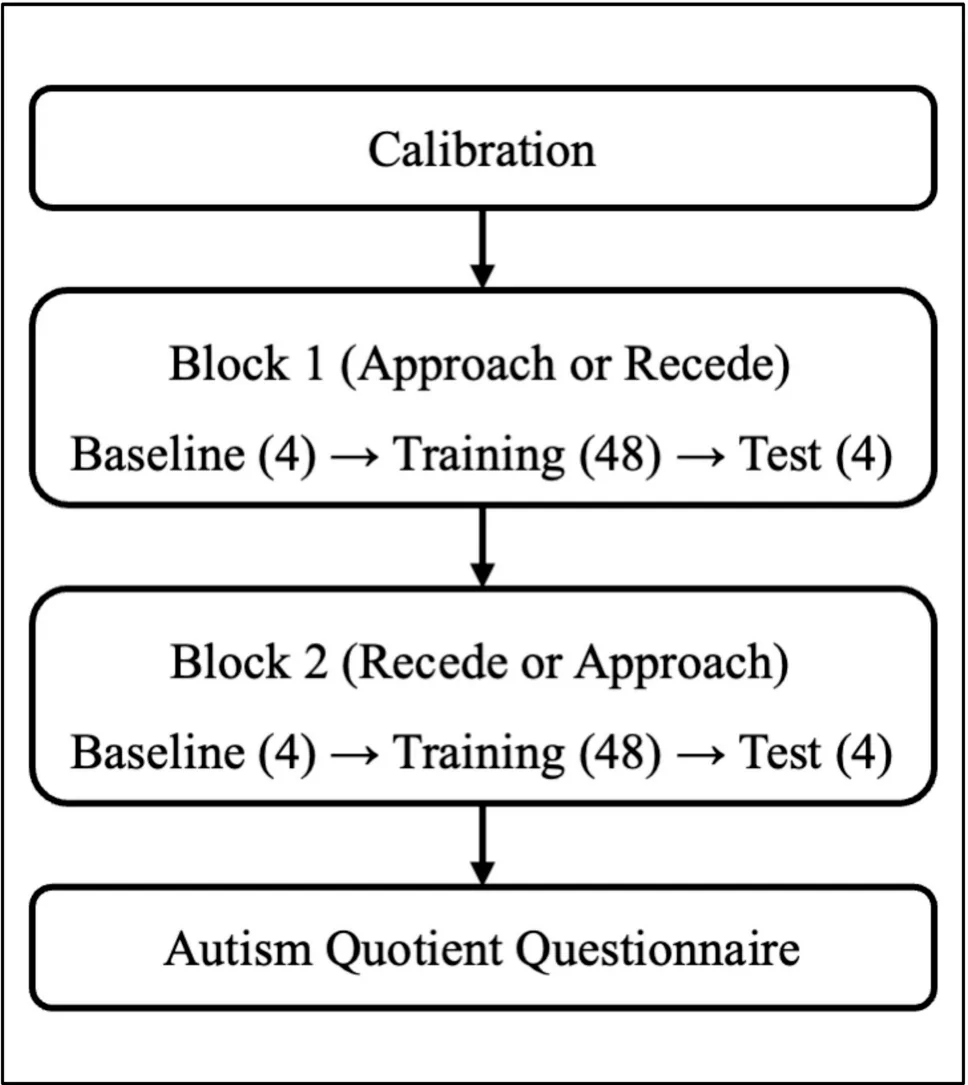
Schematic illustration of the experimental procedure

The experiment used a within-subjects design with two conditions (Approach vs. Recede) and three phases (Baseline, Training, and Test). Each participant completed two blocks corresponding to the Approach and Recede conditions. The block order was counterbalanced across participants. Before the experiment began, each participant completed a standard 9-point eye-tracker calibration procedure. Calibration accuracy was required to be below 1° of visual angle. Each block consisted of four baseline trials, 48 training trials (24 gaze-triggered, 24 static), and four test trials (Fig. 2). The number of baseline and test trials was intentionally kept small to minimize fatigue and additional exposure. Dwell-time proportions were averaged across trials and were intended to capture stable patterns of gaze allocation at the participant level. Because dwell-time proportions were averaged across trials at the participant level, the design was intended to capture stable gaze-allocation patterns while limiting additional exposure and fatigue.

After completing eye-tracker calibration, participants began the experiment without receiving any verbal instructions and without being informed of the face-outcome mappings or animation contingencies. In the baseline phase, two static faces were displayed. In the training phase, either a gaze-triggered dynamic face or a static comparison face appeared on each trial, and animations were presented only when participants fixated on the gaze-triggered dynamic face. Each training phase included 24 trials with the gaze-triggered face and 24 trials with the static face in randomized order. The identity of the gaze-triggered face and static face was fixed within each block. The Test phase, identical to the Baseline phase, followed immediately and assessed changes in gaze allocation.

After completing both experimental blocks, participants completed the Japanese version of the AQ (Wakabayashi et al., 2006).

### Analysis

Gaze points were mapped onto trial-specific areas of interest (AOIs) corresponding to each face image. The AOIs were manually defined using Tobii Pro Lab’s built-in AOI tool. Each AOI matched exactly the pixel dimensions and position of the face image on the screen. A trial was considered valid if participants’ dwell time on either face was at least 100 ms. The training phase served only as a manipulation to establish the gaze–feedback contingency and was not directly included in the outcome calculation.

The dependent variable was dwell-time proportion. This measure was defined as the proportion of dwell time directed toward the gaze-triggered face relative to the total dwell time on the two faces, and was measured in the Baseline and Test phases.

$$\mathrm{Dwell}-\mathrm{time}\ \text {proportion}=t(\mathrm{gaze}-\mathrm{triggered})/\left[t\left(\mathrm{gaze}-\mathrm{triggered}\right)+t\left(\mathrm{static}\right)\right]$$

where *t(*gaze-triggered*)* and *t(*static*)* refer to dwell times on the gaze-triggered face and static face, respectively. Dwell-time proportion in the Baseline phase reflected initial patterns of gaze allocation, whereas in the Test phase it reflected changes in gaze allocation following exposure to gaze-triggered outcomes during training. Dwell-time proportion scores were first calculated at the trial level and then averaged within each participant for each combination of Condition (Approach vs. Recede) and Phase (Baseline vs. Test). The model was therefore fitted to one averaged observation per participant for each Condition × Phase combination.

To assess whether dwell-time proportion differed as a function of gaze-triggered outcomes, we used a linear mixed-effects model to account for repeated observations across experimental conditions within participants. Dwell-time proportion was first averaged within each participant for each condition and phase, and the resulting values were entered into the model. The analysis was conducted using the lme4 package in R (version 4.3.2). Fixed effects included condition (Approach vs. Recede), phase (Baseline vs. Test), and their interaction. We also included centered AQ scores and their squared terms to test for nonlinear effects. Gender and condition order were included as covariates. Participant ID was modeled as a random effect with random intercepts and by-condition slopes. The model was fitted using restricted maximum likelihood (REML) and optimized with the *bobyqa* algorithm (maximum iterations = 200,000). Model diagnostics, including convergence checks and inspection of residual distributions, were conducted. The model converged successfully, although boundary estimates were observed in the random-effects structure. Post hoc comparisons were conducted using the emmeans package, with Bonferroni adjustments applied separately within each simple-effects family.

Analyses were divided into confirmatory and exploratory components. The confirmatory focus was the Condition × Phase interaction. AQ terms were included to test theoretically motivated individual-difference effects, whereas gender and condition order were treated as exploratory covariates.

## Results

Results are reported for dwell-time proportion as a function of Condition (Approach vs. Recede) and Phase (Baseline vs. Test), estimated using a linear mixed-effects model with AQ, gender, and block order as covariates.

A total of 50 participants were included in the analyses. The overall valid trial rate was 99.4%. Dwell-time proportion was close to chance level at baseline in both conditions (Approach: *M* = 0.52, *SD* = 0.09; Recede: *M* = 0.51, *SD* = 0.08), lower at test in the Approach condition (*M* = 0.48, *SD* = 0.11), and numerically higher in the Recede condition (*M* = 0.54, *SD* = 0.08). AQ scores ranged from 5 to 33 (*M* = 21.24, *SD* = 6.62), with only one participant scoring above the conventional cutoff of 32.

**Fig. 3:**
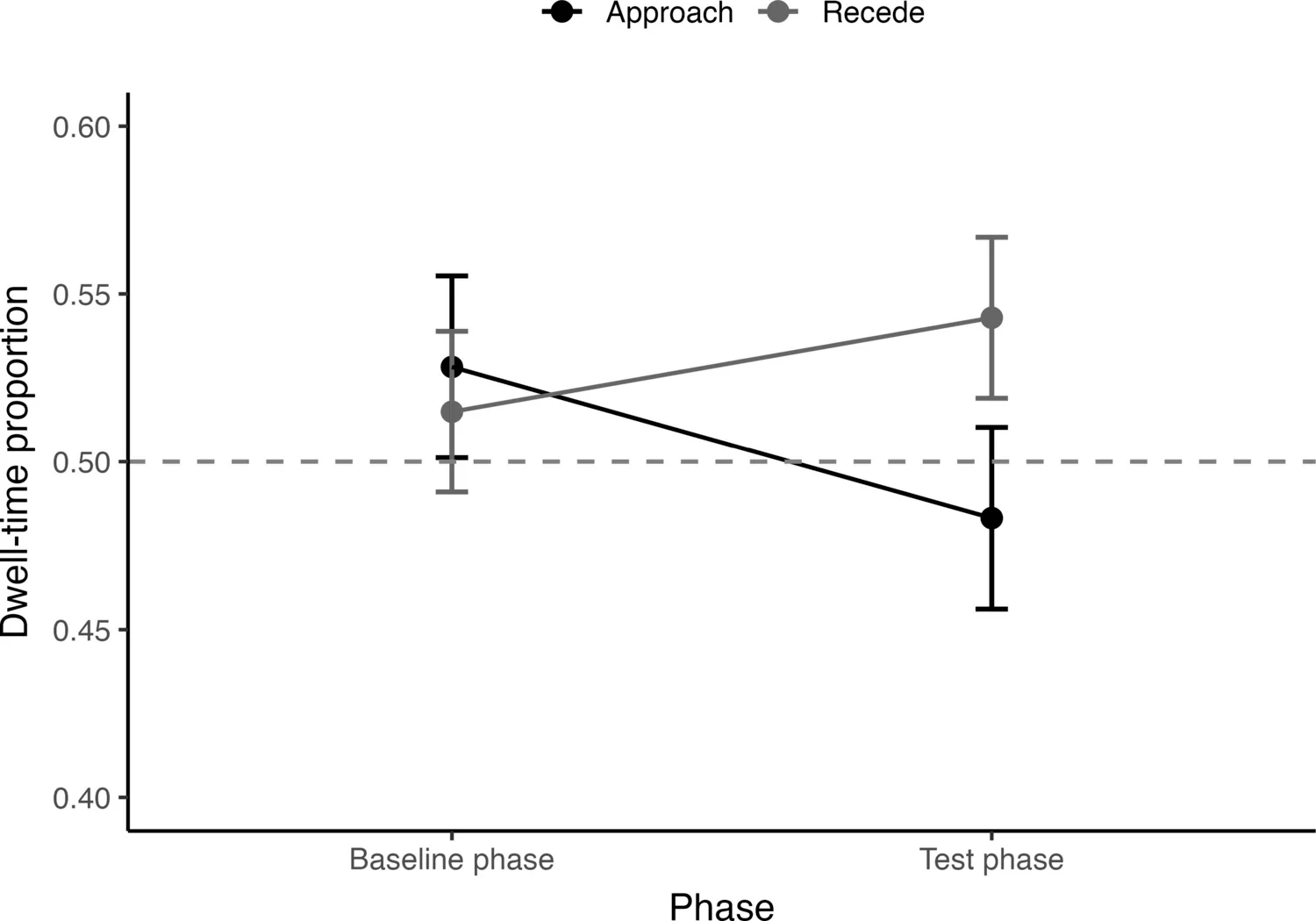
Estimated marginal means of dwell-time proportion in the Approach and Recede conditions across the Baseline and Test phases, with 95% confidence intervals

The linear mixed-effects model revealed a significant main effect of Phase, β = –0.05, *SE* = 0.02, 95% confidence interval (CI) [–0.08, –0.01], *p* = .007, indicating that dwell-time proportion was lower in the test phase than in the baseline phase. There was also a significant Condition × Phase interaction, β = 0.07, *SE* = 0.02, 95% CI [0.03, 0.12], *p* = .002 (see Fig. 3). Model diagnostics indicated successful convergence and no substantial deviations in residual distributions, based on Q–Q plots and DHARMa residual tests (see Appendix B, Fig. B1). Boundary estimates were observed in the random-effects structure, suggesting that some variance components may not have been fully supported by the data. Robustness checks using a logit-transformed outcome showed the same pattern of results (see Appendix B, Table B2), indicating that the primary findings were not driven by outcome scaling. No other fixed effects were statistically significant (all *p*s >.10). Detailed model results are presented in Table 1.

**Table 1:** Results of the linear mixed-effects model predicting dwell-time proportion

Predictor	β(Estimate)	*SE*	*df*	*t*	*p*	95% CI
(Intercept)	0.52	0.02	114.80	32.04	<.001	[0.49, 0.55]
Condition (Recede vs. Approach)	–0.01	0.02	108.20	–0.72	.474	[–0.05, 0.02]
Phase (test vs. baseline)	–0.05	0.02	101.00	–2.77	.007	[–0.08, –0.01]
AQ_total_c	0.001	0.001	47.30	0.83	.411	[–0.001, 0.003]
AQ_total_sq	0.00007	0.00012	47.30	0.59	.560	[–0.00016, 0.00031]
Gender (male vs. female)	0.02	0.01	47.30	1.67	.103	[–0.003, 0.049]
Order (first block vs. second block)	–0.02	0.01	47.30	–1.37	.178	[–0.04, 0.01]
Condition × Phase	0.07	0.02	101.00	3.18	.002	[0.03, 0.12]

AQ = Autism Spectrum Quotient, β = unstandardized regression coefficient; AQ_total_c = centered AQ total score; AQ_total_sq = squared centered AQ total score

Pairwise comparisons of estimated marginal means using the Kenward–Roger degrees-of-freedom method with Bonferroni correction indicated that dwell-time proportion significantly decreased from baseline to test in the Approach condition (estimate = 0.05, *SE* = 0.02, *t*(98) = 2.77, *p* = .013). The Recede condition showed a non-significant numerical increase (estimate = –0.03, *SE* = 0.02, *t*(98) = –1.72, *p* = .177) (see Appendix A, Tables A2–A3, for full model estimates and post hoc comparisons).

## Discussion

Results showed condition-specific changes in gaze allocation across the Approach and Recede conditions. Specifically, proportional dwell time toward the outcome-associated face decreased from Baseline to Test in the Approach condition, whereas no reliable group-level change was observed in the Recede condition, yielding a significant Condition × Phase interaction. This interaction was driven primarily by the Baseline-to-Test reduction in the Approach condition. No significant main or interaction effects involving AQ scores, gender, or block order were observed.

This pattern is consistent with the possibility that repeated exposure to gaze-triggered approach outcomes was associated with changes in subsequent gaze allocation toward the outcome-associated face. Reduced gaze allocation in this context may reflect a redistribution of attention rather than indicating subjective dislike or a stable negative evaluation of the face. The present findings highlight that the observed change in gaze allocation is compatible with context-dependent attentional modulation.

Multiple interpretations of gaze allocation should be considered. An alternative account is that reduced gaze allocation may reflect monitoring processes during training, followed by redistribution of attention when both faces were later presented as static. The present study does not allow us to conclude that gaze-triggered approach outcomes were uniquely associated with the outcome-associated face or that a specific learning mechanism underlies the observed change in gaze allocation. Because both faces were static during the test phase, the data cannot determine whether any association was attributed to the face itself or to the previously experienced gaze-triggered outcome. Therefore, we refrain from attributing the observed change uniquely to face identity effects or to outcome-history effects in the present data. Future research incorporating non-gaze-triggered control conditions may help clarify whether the observed effects are driven by the gaze-triggered structure of the task or by the size change itself. Additionally, varying facial expressions may help determine whether these effects depend on social meaning.

The present design separated gaze-triggered outcomes during training from static stimulus presentation during the Baseline and Test phases, thereby isolating subsequent gaze allocation from concurrent size-change effects. This structure allows the observed change in proportional dwell time to be interpreted as occurring in the absence of immediate stimulus motion. By pairing the same faces with gaze-triggered outcomes during training and later presenting them without motion, the paradigm was designed to detect persistent shifts in gaze allocation into the static test phase, while remaining agnostic about the underlying mechanism.

The present paradigm situates interpersonal-distance changes within the broader literature on gaze-triggered feedback across social and non-social domains. It provides a controlled framework for examining how gaze-triggered interpersonal-distance outcomes are associated with subsequent gaze allocation in the absence of concurrent motion. By distinguishing approach and recede as gaze-triggered outcomes rather than participant-initiated actions, the study contributes to clarifying how self-initiated visual behavior may be associated with later attentional allocation.

Several limitations should be acknowledged. First, because face identities were consistently paired with specific gaze-triggered outcomes during training and later presented as static in the Test phase, the design does not permit definitive separation of face identity effects from outcome-history effects. Second, the use of neutral female faces in a screen-based task may have constrained the social salience of approach and recede signals and may limit the generalizability of the findings to more naturalistic interpersonal contexts. Relatedly, because interpersonal-distance changes were implemented through changes in face size, approach and recede outcomes were necessarily accompanied by visual size changes. Although the design separated dynamic training from static test presentation, it cannot determine whether the observed changes in gaze allocation reflected processing of interpersonal-distance change, visual size change, or both. Third, although the recede condition did not produce a reliable group-level change, individual differences in the direction or magnitude of gaze allocation shifts may have contributed to the null effect.

Future research could incorporate non-gaze-triggered control conditions (e.g., randomized onset of size changes) to test whether the observed effects depend on the gaze-triggered structure rather than motion per se. It could also vary facial expressions to assess whether modulation of gaze allocation generalizes across social and affective contexts.

## Supplementary Information

Below is the link to the electronic supplementary material.

Supplementary file1 (DOCX 207 KB)

## Data Availability

De-identified trial-level data that support the findings of this study are available via the Open Science Framework at https://doi.org/10.17605/OSF.IO/EG4B6.

## Code availability

The analysis code used in this study is available via the Open Science Framework at https://doi.org/10.17605/OSF.IO/EG4B6.

## Declarations

### Preregistration

This study was not preregistered.
