# Supplementary material for: Condition-specific changes in gaze allocation following gaze-triggered interpersonal-distance outcomes
Source: Atten Percept Psychophys. 2026 Aug 31;88(7):185. doi: 10.3758/s13414-026-03322-8 (PMC13529840; doi:10.3758/s13414-026-03322-8)
Supplement: Supplementary file 1 [file 13414_2026_3322_MOESM1_ESM.docx]

Appendix A. Descriptive Statistics and Model Results

Table A1

*Descriptive statistics of dwell-time proportion by condition and phase.*

| Condition | Phase | *M* | *SD* | 95% CI |
| --- | --- | --- | --- | --- |
| Approach | Baseline | 0.52 | 0.09 | [0.50, 0.56] |
| Approach | Test | 0.48 | 0.11 | [0.46, 0.51] |
| Recede | Baseline | 0.51 | 0.08 | [0.49, 0.54] |
| Recede | Test | 0.54 | 0.08 | [0.52, 0.57] |

Note. Means and standard deviations are based on raw values (*N* = 50). 95% CIs are taken from emmeans estimates.

Table A2

*Full mixed-effects regression results predicting dwell-time proportion*

| Predictor | Estimate, β | *SE* | *df* | *t* | *p* | 95% CI | Std. β | 95% CI (Std. β) |
| --- | --- | --- | --- | --- | --- | --- | --- | --- |
| (Intercept) | 0.52 | 0.02 | 114.80 | 32.04 | < .001 | [0.49, 0.55] | 0.14 | [–0.19, 0.46] |
| Condition (recede vs. approach) | –0.01 | 0.02 | 108.20 | –0.72 | .474 | [–0.05, 0.02] | –0.15 | [–0.55, 0.26] |
| Phase (test vs. baseline) | –0.05 | 0.02 | 101.00 | –2.77 | .007 | [–0.08, –0.01] | –0.49 | [–0.85, –0.14] |
| AQ_total_c | 0.001 | 0.001 | 47.30 | 0.83 | .411 | [–0.001, 0.003] | 0.06 | [–0.08, 0.20] |
| AQ_total_sq | 0.00007 | 0.00012 | 47.30 | 0.59 | .560 | [–0.00016, 0.00031] | 0.04 | [–0.10, 0.18] |
| Gender (male vs. female) | 0.02 | 0.01 | 47.30 | 1.67 | .103 | [–0.003, 0.049] | 0.25 | [–0.05, 0.54] |
| Order (first block vs. second block) | –0.02 | 0.01 | 47.30 | –1.37 | .178 | [–0.04, 0.01] | –0.18 | [–0.44, 0.08] |
| Condition × Phase | 0.07 | 0.02 | 101.00 | 3.18 | .002 | [0.03, 0.12] | 0.80 | [0.30, 1.30] |

Note*.* β = unstandardized regression coefficient; CI = confidence interval; Std. β = standardized coefficient; AQ_total_c = centered AQ total score; AQ_total_sq = squared centered AQ total score.

Model fit: Marginal *R²* = 0.08; Conditional *R²* = 0.23.

Table A3

*Simple effects of Phase within Condition and Condition within Phase (emmeans contrasts).*

| Comparison | Estimate | SE | *df* | *t* | *p* | 95% CI |
| --- | --- | --- | --- | --- | --- | --- |
| Baseline vs. Test (Approach) | 0.05 | 0.02 | 98.00 | 2.77 | .013 | [0.01, 0.08] |
| Baseline vs. Test (Recede) | –0.03 | 0.02 | 98.00 | –1.72 | .177 | [–0.06, 0.01] |
| Approach vs. Recede (Baseline) | 0.01 | 0.02 | 108.00 | 0.72 | .948 | [–0.03, 0.06] |
| Approach vs. Recede (Test) | –0.06 | 0.02 | 108.00 | –3.22 | .003 | [–0.10, –0.02] |

Note. Simple-effects contrasts of the estimated marginal means were conducted using the Kenward–Roger degrees-of-freedom method. Bonferroni adjustments were applied separately to two families of comparisons: Phase contrasts within each Condition and Condition contrasts within each Phase. The table reports non-standardized contrast estimates and Bonferroni-adjusted 95% confidence intervals.

Appendix B

*Model Diagnostics and Robustness Checks*

Figure B1. Residual diagnostics for the linear mixed-effects model. The left panel shows the quantile–quantile plot of standardized residuals; the right panel shows DHARMa residuals plotted against model predictions. No significant deviations were detected (Kolmogorov–Smirnov, dispersion, and outlier tests all nonsignificant). Red asterisks indicate boundary residuals.

Table B2

*Full mixed-effects regression results predicting logit-transformed dwell-time proportion*

| Predictor | Estimate, β | *SE* | *df* | *t* | *p* | 95% CI |
| --- | --- | --- | --- | --- | --- | --- |
| (Intercept) | 0.09 | 0.07 | 123.40 | 1.26 | .211 | [–0.05, 0.22] |
| Condition (recede vs. approach) | –0.05 | 0.08 | 107.90 | –0.66 | .513 | [–0.21, 0.10] |
| Phase (test vs. baseline) | –0.19 | 0.07 | 143.50 | –2.79 | .006 | [–0.32, –0.06] |
| AQ_total_c | 0.004 | 0.004 | 98.20 | 0.86 | .393 | [–0.004, 0.011] |
| AQ_total_sq | 0.0003 | 0.0005 | 98.20 | 0.63 | .530 | [–0.0007, 0.0013] |
| Gender (male vs. female) | 0.10 | 0.06 | 98.20 | 1.68 | .096 | [–0.01, 0.20] |
| Order (first block vs. second block) | –0.07 | 0.05 | 98.20 | –1.35 | .180 | [–0.17, 0.03] |
| Condition × Phase | 0.31 | 0.10 | 143.50 | 3.18 | .002 | [0.12, 0.49] |

Note. The restricted maximum likelihood (REML) criterion was 204.60.
